# Analysis of the Relationship Between Scale Invariant Feature Transform Keypoint Properties and Their Invariance to Geometrical Transformation Applied to Cone-Beam Computed Tomography Images

**DOI:** 10.3390/bioengineering11121236

**Published:** 2024-12-06

**Authors:** Diletta Pennati, Leonardo Bocchi

**Affiliations:** 1 Department of Information Engineering, University of Florence, 50139 Florence, Italy; diletta.pennati@unifi.it; 2 EIDO Lab, University of Florence, 50139 Florence, Italy

**Keywords:** cone-beam computed tomography, keypoint detection, scale-invariant feature transform, image registration

## Abstract

Image registration is a crucial post-processing technique in biomedical imaging, enabling the alignment and integration of images from various sources to facilitate accurate diagnosis, treatment planning, and longitudinal studies. This paper explores the application of Scale Invariant Feature Transform (SIFT), a robust feature-based method for the alignment of biomedical images. SIFT is particularly advantageous due to its invariance to scale, rotation, and affine transformations, making it well-suited for handling the diverse and complex nature of biomedical images. However, SIFT was not initially developed specifically for medical imaging applications, so it is necessary to adapt the algorithm to those kinds of images. In particular, this work was focused on images obtained with Cone-Beam Computed Tomography (CBCT) technology. Besides fine-tuning SIFT parameters on a case-by-case basis, the novelty of this work consists of finding the optimal SIFT parameters on the basis of the keypoints stability. A statistical analysis throughout a dataset of images obtained with CBCT technology was performed to find the best SIFT parameters setting, in terms of computational cost and result quality, compared to default presets.

## 1. Introduction

Cone-Beam Computed Tomography is a non-invasive imaging technique that provides 3D high-resolution images of the human body, enabling detailed visualization of anatomical structures [1]. However, the use of CBCT in medical imaging is not without challenges. One of these is image registration, which can be performed both at 2D level (single slices registration) and at 3D level (volumetric registration). Traditional methods often rely on manual or semi-automatic processes, which are time-consuming, operator-dependent, and consequently prone to errors. Therefore, there is a need for automated methods that can accurately register 2D slices and 3D volumes [2]. In the clinical field, several applications require accurate and efficient image registration. For example, it can be used in pre- and post-contrast images, where anatomical structures remain the same, but are not perfectly aligned between the scans before and after the contrast agent injection. Instead, in pre- and post-operative scenarios, image registration is a crucial part of the process that enables consistent and comparable visualization of the patient’s anatomy before and after surgery. This is essential for diagnosis, treatment planning, surgical guidance and follow-up. This application is more challenging compared to the previous one, as the datasets are acquired at considerably different times; therefore, the discrepancies between the volumes to be aligned will be a combination of translation, rotation and scaling. Image registration can also be applied in multimodal applications if the aim is to find correspondences between studies of different natures (CBCT and Magnetic Resonance Imaging (MRI) or CBCT and Single Photon Emission Computed Tomography (SPECT)). For this purpose, feature-based methods achieve better results than intensity-based ones because grayscale correspondence is not guaranteed when dealing with different modalities. Instead, detecting similar anatomical characteristics using keypoints methods is more promising. Several methods were studied in the past years, both intensity and feature-based. In the first case, voxel properties are considered, which certainly can vary among datasets acquired with the same imaging modality [3]. A promising approach to this challenge is the use of keypoint-based methods, which are an extension of the SIFT algorithm to 3D volumes. The SIFT algorithm is a popular method for detecting and describing local features in two-dimensional images, developed by D.G.Lowe [4]. This approach transforms image data into scale-invariant coordinates relative to local features generating a large number of features that densely cover the image over the full range of scales and locations. Two-dimensional image registration can be used for several applications in the medical field. One of these is the alignment of overlapping slices between consecutive scans of the same volume. If the system is well designed and the patient is cooperating, it is possible to assume the coplanarity among consecutive scans, thus registration can be performed slice by slice, obtaining a reliable 3D registration starting from 2D slices, as shown in [5], where the method has been validated by applying a known transformation matrix to a set of CBCT data. If the coplanarity between slices cannot be assumed, the extension to 3D volumes may provide a robust and efficient method for 3D volume registration [1] based on the features detection directly in the 3D volume. For instance, a study by Zhou et al. [2] demonstrates the use of a keypoint-based method for registering 3D volumes of the condyle and skull base. The study shows that the method can effectively correct large deviations avoiding local minima, leading to accurate and reliable registration results. Another study by Weissheimer et al. [6] uses a keypoint-based method to register different CBCTs at the anterior cranial base. The method is able to superimpose the 3D volumes accurately, demonstrating its potential for use in medical imaging. However, all of these methods emphasize the fact that registration performed in the 3D space has a high computational cost and requires small transformations between the reference volume and the volume to be aligned.

In this paper, we investigate the possibility to define a criterion for selecting only the keypoints that contribute to the final correct alignment may ameliorate both issues. In particular, we analyze the optimal values of the different thresholds—specifically contrast and eigenvalue ratio thresholds—that distinguishes real keypoints from the other candidate pixels inside the SIFT algorithm. In the previously cited works [1,2,6], these thresholds are not defined with a specific criterion, but they are fine-tuned depending on the current application.

A statistical analysis has been performed starting from the 2D case over a set of CBCT slices with a known transformation applied, in order to demonstrate SIFT robustness to transformation changes. The selection of the datasets and of the applied transformations are presented in Section 2. This investigation aims to find a correlation between the already mentioned SIFT threshold and the keypoint stability to various initial applied transformation matrices.

The results will be presented in Section 3. Through this statistical analysis, the optimal threshold values were identified, enhancing keypoint stability and reducing the algorithm computational cost, without compromising the registration accuracy. Finally, in Section 4, we will underline that this optimization allows more efficient image alignment by using fewer, more stable keypoints, which can benefit diagnostic and treatment planning processes in medical imaging.

## 2. Materials and Methods

A set of six different CBCT datasets representing various anatomical districts was used for this work. All of these datasets belong to veterinary patients, and they were acquired following all the applicable international, national, and institutional guidelines for the care and use of animals. Moreover, the images were not generated for the scope of the study, but they were previously acquired and stored from different veterinary clinics.

The datasets have different sizes and were acquired with three different DICOM resolutions (0.25 mm, 0.35 mm and 0.6 mm) (Table 1). A set of sample slices was randomly extracted from each dataset; then, slices that appeared similar were discarded, in order to have a collection of images as different as possible, thus obtaining a set of 27 images $I_{k}$. We randomly generated a set of 12 affine transformation matrices $T_{i}\left(.\right)$, i.e., including not only simple translations, rotations and scaling, but also a combination of them (see Table 2). Applying all transformations to each image, we obtained 324 images $T_{i}\left(I_{k}\right)$ that participated in the statistical analysis. First of all, keypoints $K_{k}$ were detected using SIFT algorithm in the original image $I_{k}$ and counted. Then, keypoints $K_{k,j}$ were also extracted from each transformed image $T_{i}\left(I_{k}\right)$. To verify how many identical features were detected in the original and in the deformed image, transformed keypoints $T_{i}\left(K_{k}\right)$ were obtained applying the transformation matrix to reference ones; we considered a keypoint is correctly detected in both images if the distance between $T_{i}\left(K_{k}\right)$ and $K_{k,j}$ is smaller than a predefined threshold (set to 5 pixels).

### 2.1. Keypoints Detection

The keypoint detection method used in this work is based on the already mentioned SIFT algorithm, developed by D.G. Lowe. A brief description of SIFT process is provided in this paragraph to better understand the meaning of the threshold fine-tuning. However, for a more complete description of the methods, the original work [4] should be used as a reference.

SIFT uses a so-called multi-resolution approach, which permits the identification of only those features that are representative of the analyzed images and that are thus recognizable at different resolution levels.

- Scale-space representation: The image is first convolved with Gaussian filters at different scales, creating a scale-space representation. This is performed by generating progressively blurred versions of the original volume, then resizing the original image to half its size and generating blurred versions again. These representations are grouped by octaves, which correspond to doubling the value of the scale. The adjacent scales within an octave differ by a constant factor *k*, which depends on the number of intervals *s* between octaves. According to D.G. Lowe, this value is equal to $k=2^{1/s}$ [4].
- Difference of Gaussians (DoG) calculation: The difference of successive Gaussian-blurred levels are taken. This results in a series of DoG images D(x,y,z,σ). A DoG image between scales $k_{i}\sigma$ and $k_{j}\sigma$ is just the difference of the Gaussian-blurred images at scales $k_{i}\sigma$ and $k_{j}\sigma$.
- Keypoint detection: The keypoint candidates are assumed to be local extrema (maxima or minima) in the DoG scale space. To achieve this, each sample point is compared with 26 neighbors, including 8 neighbors in the current volume and 9 neighbors each in the upper and lower scales. A candidate keypoint is selected only if it is greater than all adjacent keypoints or less than all keypoints [7]. According to Rister et al., local extrema are defined using the 2(n+1)-connected $L^{1}$ neighborhood (*n* = image dimensionality; in this case n=2), instead of the ($3^{n+1}-1$)-connected $L^{\infty }$ neighborhood. While this results in a considerable increase in the number of extrema, and thus the necessary computation, it yields a far greater number of correctly matched keypoints [8].
- Keypoint refinement: Since some of the keypoint candidates are unstable, a detailed fit to the nearby data for accurate location, scale, and ratio of principal curvatures is performed. This information allows the rejection of points that are low contrast (and are therefore sensitive to noise) or poorly localized along an edge.
- Orientation assignment: By assigning a consistent orientation to each keypoint based on local image properties, the keypoint descriptor can be represented relative to this orientation and therefore achieve invariance to image rotation. For each Gaussian smoothed image sample, the gradient magnitude, m(x,y), and orientation, θ(x,y), are precomputed using pixel differences. An orientation histogram is formed from the gradient orientations of sample points within a region around the keypoint. The orientation histogram has 36 bins covering the 360-degree range of orientations. Each sample added to the histogram is weighted by its gradient magnitude and by a Gaussian-weighted circular window. Peaks in the orientation histogram correspond to the dominant directions of local gradients.
- Descriptor generation: Descriptors encode information about a keypoint’s neighborhood and allow comparison between keypoints. We consider again a square neighborhood around each keypoint, rotated by the keypoint’s angle. This is what makes SIFT invariant to rotation. An analogue Gaussian-weighted histogram is calculated, but this time, all neighbor histograms are concatenated to build the descriptor vector.

### 2.2. Keypoint Stability

**Figure 1:**
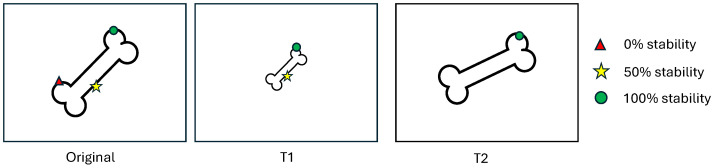
Explanation of what keypoints stability means: considering the original image and two different deformed versions, only keypoints that appear in all of the three images can be considered 100% stable.

To measure the keypoint strength, a parameter called stability has been defined. As shown in Figure 1, the stability expresses the percentage of success in finding the same keypoint both in the original image and in the transformed one. A stability equal to 100% means that the keypoint can be found whatever the transformation is; therefore, it can be considered a good representative point for the image. Based on this parameter, the idea of this work is to identify the keypoint with the higher stability and use only these to perform the alignment between two images, thus discarding the weaker ones, according to the stability definition. Since the transformation matrix that relates the reference image to the one to be registered is not known (it is the unknown variable indeed), it is not possible to calculate the keypoint stability directly; thus, we aim to find a relationship between stability and keypoint features.

### 2.3. SIFT Thresholds

As previously said, SIFT method has been used to detect keypoints. By analyzing the algorithm, it is possible to notice that keypoints are selected from candidate extrema pixels according to two thresholds:

- Contrast threshold: unstable extrema with low contrast are rejected if their intensity, normalized in the range [0, 1], is lower than a certain threshold, fixed to 0.04.
- Eigenvalues ratio threshold: For stability, it is not sufficient to reject keypoints with low contrast. The difference-of-Gaussian function will have a strong response along edges, even if the location along the edge is poorly determined and therefore unstable to small amounts of noise. A poorly defined peak in the difference-of-Gaussian function will have a large principal curvature across the edge, but a small one in the perpendicular direction. The principal curvatures are proportional to the eigenvalues of the Hessian matrix. We can avoid explicitly computing the eigenvalues, as we are only concerned with their ratio. Keypoints whose eigenvalue ratio is above a certain threshold (fixed to 10) are discarded because they poorly define edges, according to the definition in the previous lines.

By fine-tuning these parameters, it is possible to reduce or augment the number of final keypoints that are used for matching. If a statistically significant correlation between keypoints stability and these SIFT parameters exists, we can adjust the threshold in order to keep only more stable keypoints.

### 2.4. Statistical Analysis

For each keypoint found in the original slices from the 6 datasets and their transformed version, the following parameters were saved:

- Keypoints coordinates, for checking the correspondences between reference and deformed images. To avoid eventual errors resulting from bringing back the coordinate from the octave (i.e., level of downsampling), where the keypoint has been detected, to the original image size, the coordinates check is performed directly at the octave level.
- Contrast and eigenvalue ratio values: Each keypoint has associated these two parameters, which are obviously within the imposed threshold.
- Stability rate: for each keypoint in the original image, the number of occurrences is counted and divided by the number of considered transformations. For example, if a keypoint has been detected in 6 out of 12 transformations, its stability rate is equal to 0.5.

Therefore, the data to be correlated are the contrast and eigenvalue ratio on one side and the stability rate value on the other side, associated with each keypoint detected in each image with each transformation matrix applied. To establish if the data are normally or non-normally distributed, a Shapiro–Wilk test (if the number of elements is less than 5000) or a Kolmogorov–Smirnov test (n>5000) is performed. The variables were statistically described as mean ± standard deviation (SD) for normally distributed quantitative data; median and interquartile range (IQR) for non-normally distributed data. Then, the data correlation is calculated using Spearman or Pearson test (for Gaussian and non-Gaussian distributions, respectively). The significance level for all the tests was set to $p_{value}<0.05$. The null hypothesis $H_{0}$ of the test is that no correlation exists among variables; when $p_{value}<0.05$, $H_{0}$ will be rejected, thus the variables are correlated, otherwise it is not possible to reject $H_{0}$, so the correlation is not statistically significant.

## 3. Results

**Figure 2:**
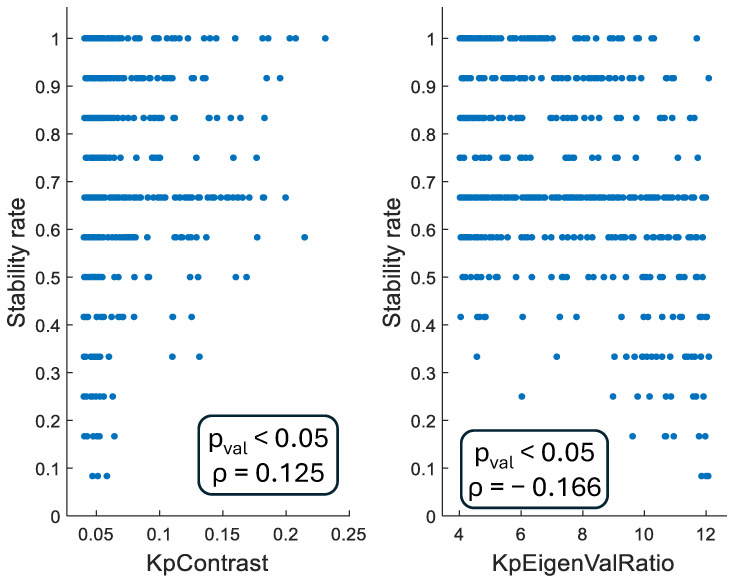
Scatter plots of stability rate against keypoint contrast (**left**) and keypoint eigenvalues ratio (**right**) demonstrating the statistically significant positive and negative monotonic relationship, respectively, among variables.

The evaluation of the relation between stability rate and the algorithm parameters involves all keypoints in the original dataset and their transformed version, totaling 4785 keypoints. Figure 2 shows that a statistically significant relationship has been found both between stability rate and keypoint contrast (ρ=0.125, $p_{value}<0.05$) and between stability rate and keypoint eigenvalue ratio (ρ=−0.166, $p_{value}<0.05$). The sign of the correlation coefficient ρ is in agreement with the two threshold meanings. Increasing values of the contrast parameter are associated with the higher the stability of that keypoint (positive monotonic relationship), concluding that keypoints closer to the threshold are less stable than those quite larger than the threshold itself. Therefore, a larger contrast threshold helps to reject the keypoints having a small stability. On the other hand, the lower the eigenvalue ratio parameter, the higher the stability of that keypoint (negative monotonic relationship). Therefore, using the same argument, a lower contrast threshold than the default one improves the probability of selecting the most stable keypoints.

Based on this result, the next objective of this work is to assess the improvement on the registration task obtained by optimizing the contrast and eigenvalue ratio thresholds. It is evident that by using more strict thresholds, a lower number of keypoints will be detected. However, according to the previously described correlation, we expect that the stability of the selected keypoints increases and, therefore, the number of well matched keypoints should also increase.

For each value of contrast and eigenvalue ratio thresholds, the keypoint detection has been carried out in each pair of images (original and transformed one). In every image, we imposed to select a minimum number (at least 10) of keypoints: this permitted us to avoid cases where the number of keypoints is too small, which could inexorably alter the final registration.

Detected keypoints are matched through a Euclidean-distance-based nearest-neighbor approach. According to Lowe’s work, matches are rejected for those keypoints for which the ratio of the nearest neighbor distance to the second-nearest neighbor distance is greater than a certain value (generally ranging from 0.8 to 0.9) [4]. To increase matching robustness, another approach was used in this work, based on a cross-check between the two images to be aligned. This consists of a knn-Match method with k=1 that will only return consistent pairs (i,j) such that, for the i-th query descriptor, the j-th descriptor in the matcher’s collection is the nearest, and vice versa. Such a technique usually produces the best results with the minimal number of outliers when there are enough matches.

The matched keypoint pairs constitute the input for the evaluation of the optimal limited affine transformation, with four degrees of freedom, that transforms the first element of the pairs into the second one (representing the transformation of the matched keypoints in the two images).

The quality of the final alignment has been evaluated by calculating the root mean square error (rmse) of the difference image between the original image deformed using the affine transformation matrix and the test image.

**Figure 3:**
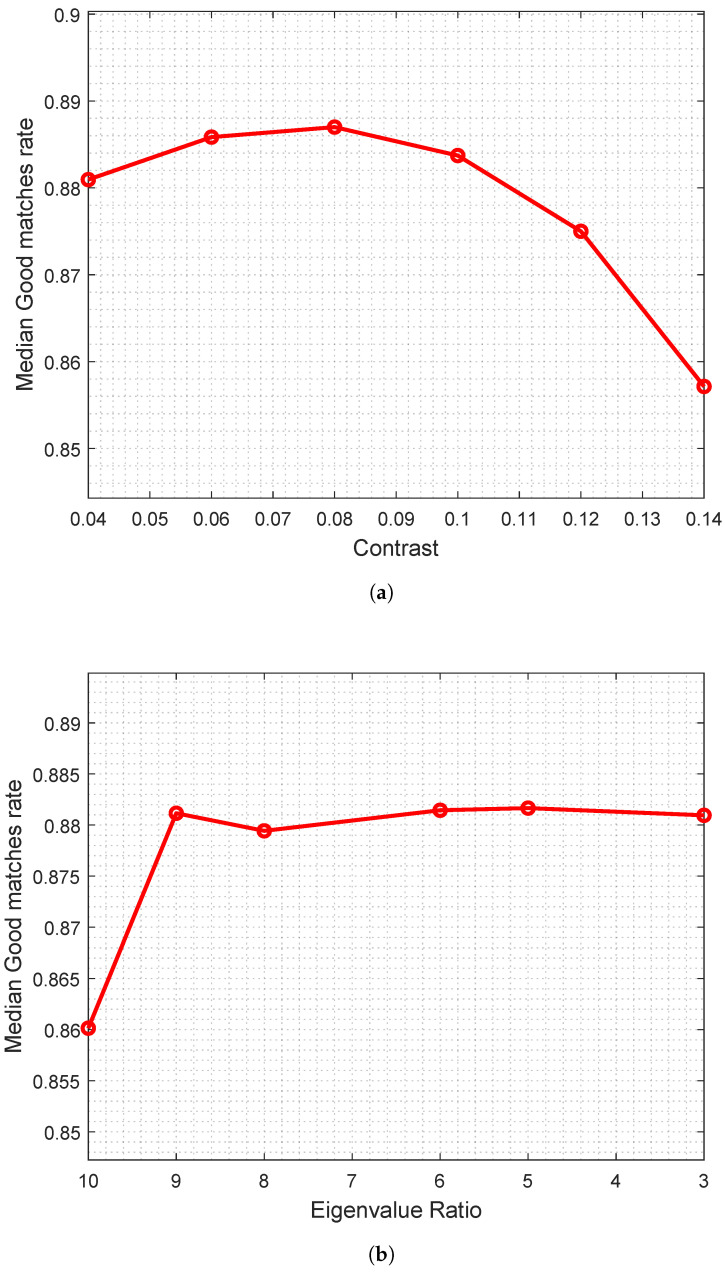
(**a**) Median trend of correct matches at varying contrast values. The algorithm shows the best performances at c = 0.08. (**b**) Median trend of good matches at varying eigenvalue ratio values. The algorithm shows a sudden rise of the percentage of good matches and than remains quite constant.

**Figure 4:**
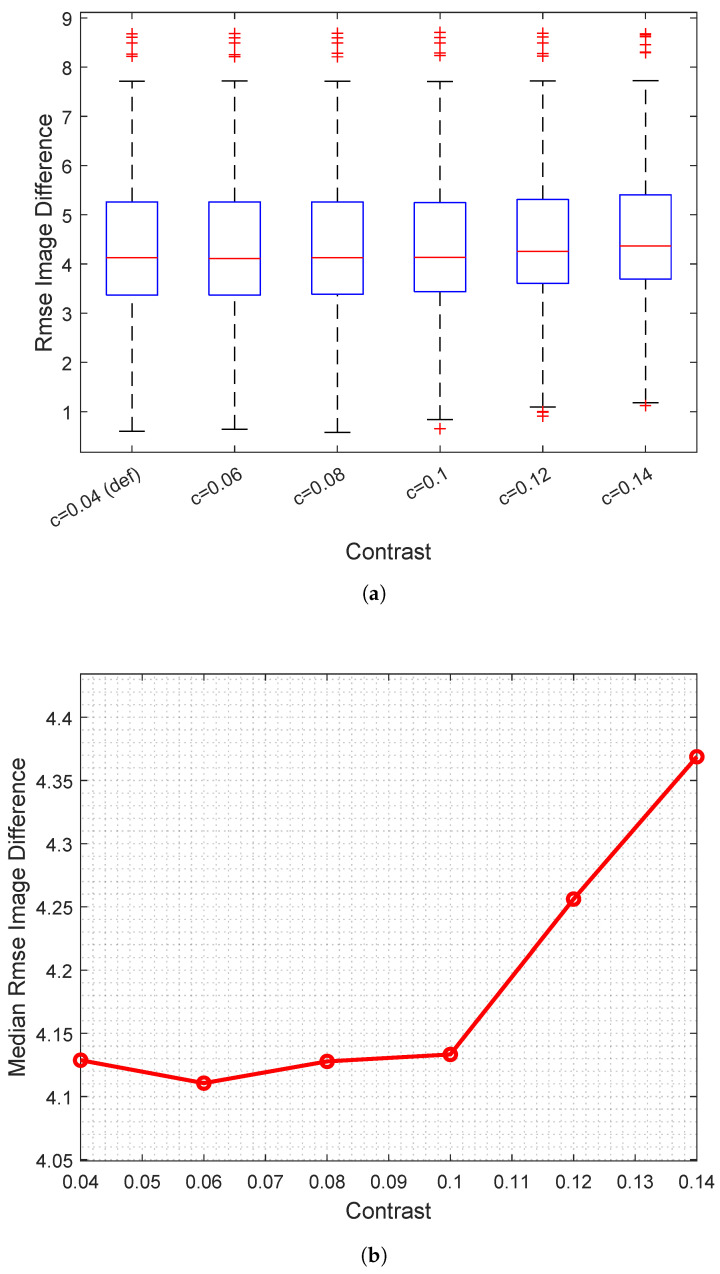
(**a**) Box plots representing the distribution of the root mean square error of the resulting difference image after the registration at variable contrast thresholds. (**b**) Focusing on the median trend, it is possible to notice that until c = 0.1 the rmse is approximately stable, wile for larger values the accuracy decreases.

**Figure 5:**
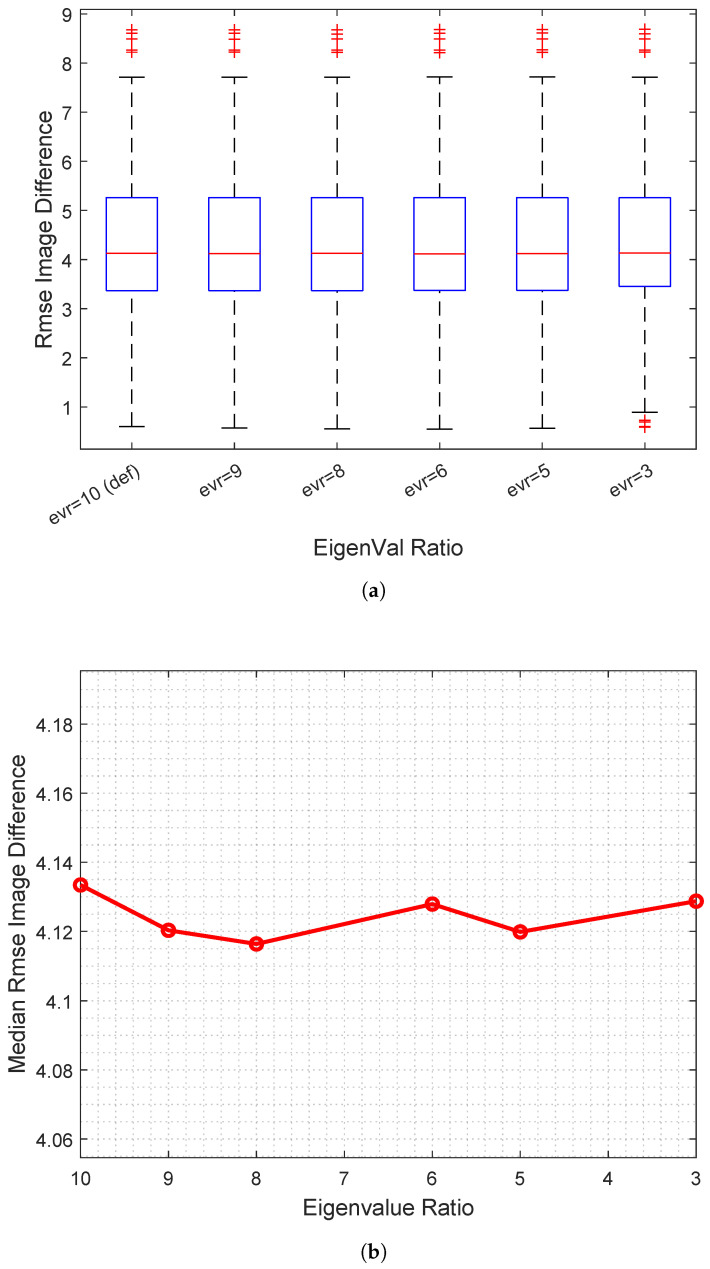
(**a**) Box plots representing the distribution of the root mean square error of the resulting difference image after the registration at variable eigenvalues ratio thresholds. (**b**) Focusing on the median trend, it is possible to observe that there are no substantial differences in the resulting output if the threshold is reduced. This suggests that the keypoints discarded using a lower threshold do not significantly participate in the correct output registration.

Figure 3, Figure 4 and Figure 5 show the results. First of all, an increase in the median good matches rate has been observed with respect to the default threshold values (Figure 3). From a certain value on, the trend remains constant or slightly decreases; this event is due to the fact that the detected keypoint number drops as the threshold becomes more restrictive; therefore, there is also a lower probability of having a higher number of good matches. At first sight of the boxplots in Figure 4a and Figure 5a, it is possible to notice that the distribution of the rmse of the difference image is quite similar at varying contrast and eigenvalue ratio values.

Examining the median rmse trend more in detail (Figure 4b and Figure 5b), it is evident that there is a minimum in 0.06 and 8 in contrast and eigenvalue ratio graph, respectively, then the error starts to increase again for the same reason explained for the good matches rate trend.

**Figure 6:**
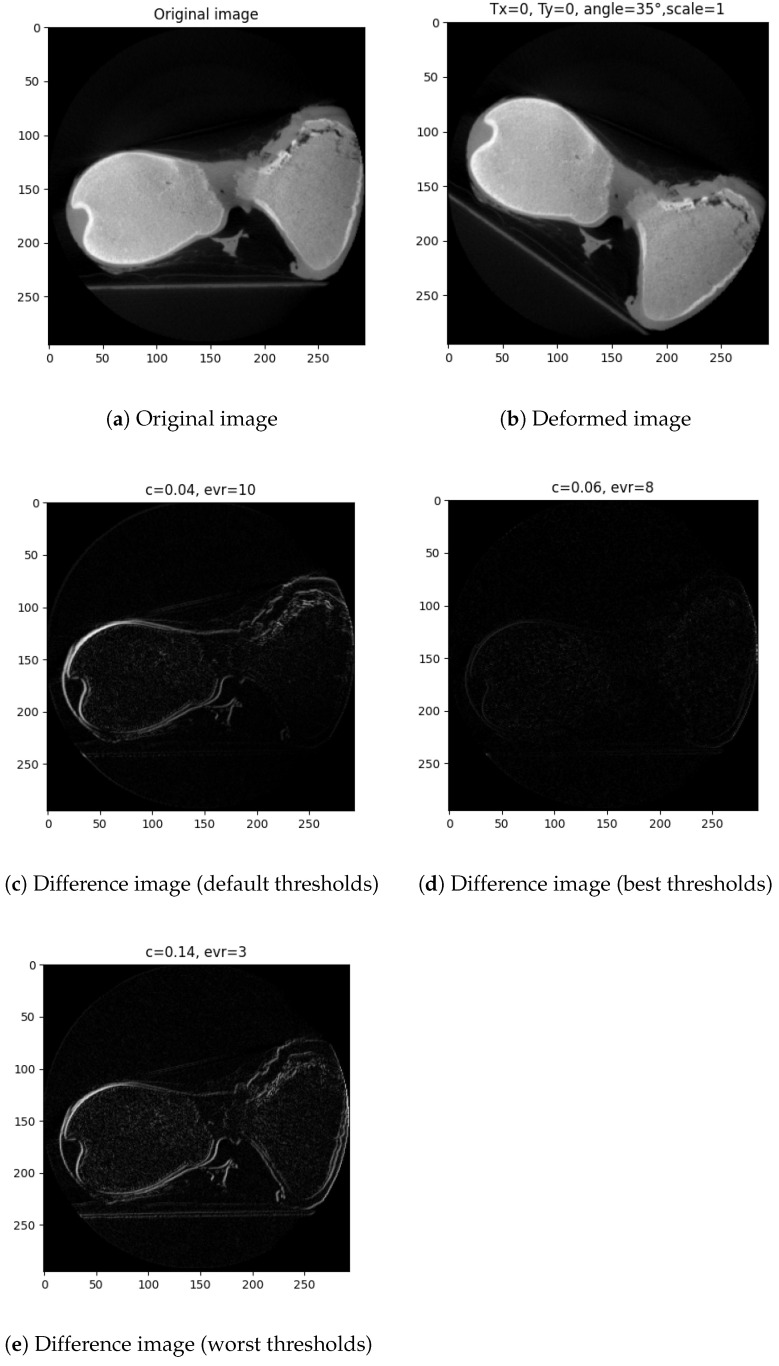
Example of the result of the registration between reference (**a**) and deformed image (**b**), shown using the difference image. In (**c**), the output registration matrix was calculated using the default SIFT thresholds (c = 0.04, evr = 10); in (**d**), the output obtained with the thresholds that better performed, according to Figure 4 and Figure 5 (c = 0.06, evr = 8); in (**e**), the output obtained with the thresholds that gave the higher rmse in image difference (c = 0.14, evr = 3).

In Figure 6, an example of images alignment obtained using different SIFT thresholds is presented. In particular, Figure 6a,b are the reference and deformed image, respectively. Figure 6c is the registration output, expressed as difference image, obtained using default SIFT thresholds (c=0.04, evr=10). Instead, Figure 6d the output obtained with the thresholds that better performed, according to Figure 4 and Figure 5, corresponding to c=0.06 and evr=8. Finally, Figure 6e the output was obtained with the thresholds that gave the higher rmse in image difference, according to Figure 4 and Figure 5, corresponding to c=0.14, evr=3). As expected, the best result was obtained using the thresholds that minimize the rmse of the image difference, as highlighted with the statistical analysis.

**Figure 7:**
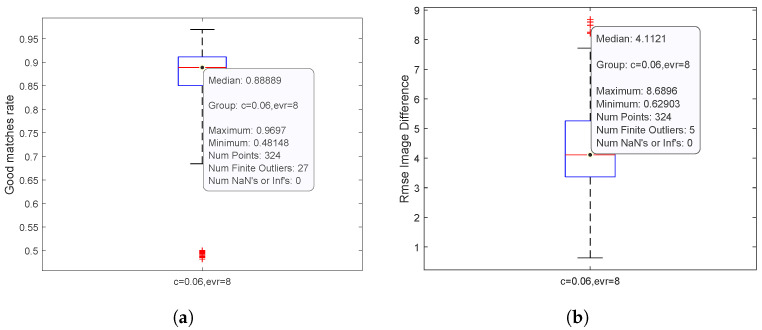
Good match rates (**a**) and rmse of the difference image (**b**) box plots using best contrast and eigenvalue ratio thresholds.

At this point of the analysis, the best thresholds resulted from the previous analysis were combined and the results in terms of good matches rate and rmse of the difference image were collected. As shown in Figure 7, the median values are aligned to those calculated considering the two parameters separately, so it is not possible to conclude that there is a cumulative effect of changing both thresholds.

**Figure 8:**
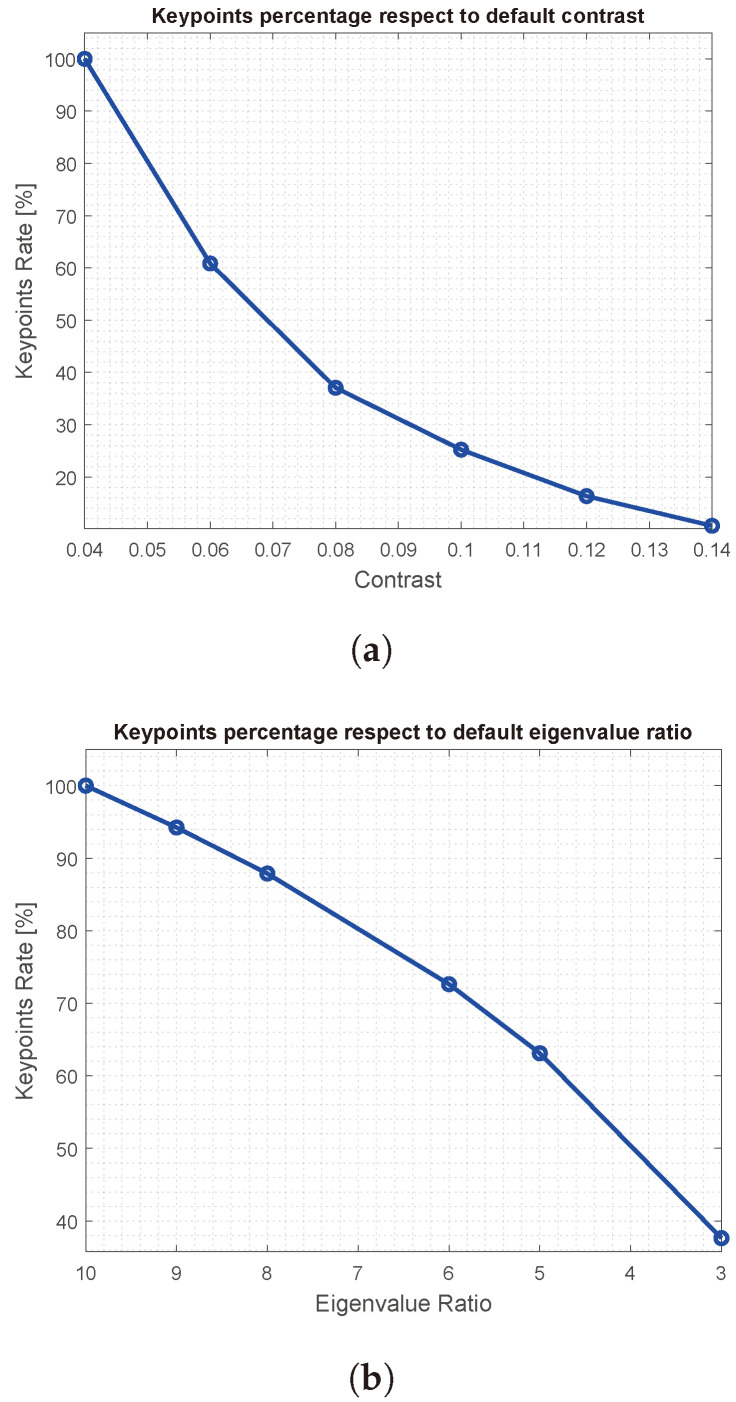
Keypoints number trend using different SIFT thresholds, normalized respect to the keypoints amount detected with the default values. (**a**) Varying the contrast threshold, on the basis of the results shown in Figure 4, it is possible to discard about 50% of the keypoints to obtain the same result as in the original case. (**b**) Regarding the variation in the eigenvalue ratio threshold, to obtain the best result in terms of image difference rmse (see Figure 5), about 80% of the keypoints are needed.

The correlation between the SIFT thresholds and the keypoint stability suggests that keypoints closer to the default thresholds are less stable and more sensitive to an eventual transformation applied to the original image. This argumentation brings to the idea that these weaker keypoints do not contribute significantly to the output transformation matrix, so it is reasonable to discard them using a different threshold. In Figure 8, it is possible to observe the variation in the number of detected keypoints compared to the amount obtained with the default thresholds.

Summing up the results, it is possible to conclude that a doubling of the contrast threshold (from 0.04 to 0.08) and a halving of the eigenvalue ratio threshold do not change the registration quality, as highlighted in Figure 4 and Figure 5. Combining the information from Figure 4, Figure 5 and Figure 8, it is possible to conclude that less than 50% of the keypoints are needed for obtaining the same quality as with the original thresholds.

As well as obtaining a more accurate final registration, threshold refinement permits speeding up SIFT algorithm in keypoint detection. In fact, for each keypoint, a descriptor must be calculated for the following matching. This step is one of the most time consuming, because a new analysis of the image gradient magnitudes and orientations sampled around the keypoint location must be performed. An evaluation of time efficiency increase has been performed. For example, using the optimal thresholds resulting from the statistical analysis (0.06 and 8 for contrast and eigenvalue ratio, respectively), a decrease of 20% of time needed for completing the entire algorithm was observed.

## 4. Discussion

The results of this study highlight the significant correlation between the SIFT keypoint stability and its associated contrast and eigenvalue ratio thresholds, offering an avenue for optimizing image registration processes in medical imaging. This optimization leads to a reduction in computational cost while maintaining and, in some cases, improving the accuracy of registration outcomes. These findings are considerably promising for real clinical applications, where efficiency and reliability are fundamental.

In the literature, several studies have been published on SIFT comparison with other keypoint detection techniques [9,10,11], but none of them have focused on the optimization of algorithm thresholds. Moreover, when SIFT is used for biomedical images, these parameter adjustments are made without any specific criteria. Instead, the results of this work suggest that it is possible to define a criterion to keep only the keypoints that really contribute to the correct final registration matrix.

The demonstrated ability to use fewer and more stable keypoints has several practical implications. First, this refinement can enable faster processing of image datasets, which is critical in time-sensitive scenarios, such as surgical navigation and real-time diagnostics. Furthermore, the improved computational efficiency promotes the integration of SIFT-based methods into resource-constrained environments, where computational performing hardware may not be available.

Optimized keypoint selection could significantly enhance workflows in multimodal imaging, where CBCT is used alongside other imaging techniques, such as MRI or SPECT scans. Accurate alignment across modalities could improve diagnostics by enabling a comprehensive visualization of anatomical structures and pathological conditions. In pre- and post-operative evaluations, the ability to achieve more efficient and precise image registration could enhance the planning and assessment of surgical interventions, potentially leading to better patient outcomes.

In research settings, this method could streamline large-scale studies involving volumetric image datasets, such as longitudinal studies on disease progression or multi-patient comparative analyses. The reduced processing time would allow researchers to process more datasets simultaneously.

While the study demonstrates the advantages of threshold optimization, some challenges remain. For example, the selection of thresholds requires an initial statistical analysis, which may not be feasible for institutions lacking access to comprehensive datasets. Furthermore, the approach assumes that keypoint stability is the most critical factor influencing registration accuracy. However, other factors, such as image noise, artifact presence, or variations in scanner calibration, could also affect the results and warrant further investigation. Moreover, other statistical analysis on not X-ray-generated images should be carried on to find out the optimal thresholds for each involved imaging modality.

As mentioned in the introduction, SIFT method can also be extended to 3D to perform volume registration [7,8]: therefore, this analysis could be extended to volumetric datasets, where both the quality and the efficiency increase are fundamental for a correct registration, to find the best thresholds and optimize the keypoint number for obtaining an acceptable alignment.

Additionally, integrating machine learning techniques to dynamically adjust SIFT thresholds based on dataset characteristics could enhance adaptability and automation. This would eliminate the need for extensive upfront statistical analysis, making the method more accessible for routine clinical use.

Exploration of hybrid registration techniques, combining optimized SIFT with intensity-based or neural network-based methods, could also yield further improvements in both accuracy and robustness, particularly for challenging multimodal or noisy datasets.

## 5. Conclusions

This work has focused on finding out a correlation between some SIFT parameters used to detect keypoints and the keypoint stability itself. The statistical analysis was conducted on CBCT datasets, from which sample slices were extracted to obtain 2D images. These images were deformed using a known transformation matrix, which we need to verify that the output registration has performed as expected.

The findings highlight the importance of tailoring general-purpose algorithms, such as SIFT, to the specific needs of biomedical imaging, challenging the reliance on default parameter settings. This optimization not only enhances performance but also reduces the resource demands, making the approach feasible for a wider range of imaging environments, including those with constrained computational capabilities.

While the results are promising, future work should extend this analysis to 3D volumetric datasets and other imaging modalities to validate the approach’s broader applicability. Additionally, integrating this method with machine learning and hybrid registration techniques could unlock further potential, paving the way for highly efficient, automated solutions in biomedical image processing.

## Figures and Tables

**Table 1:** Datasets characteristics used for extracting sample images.

Dataset ID	Image Size [px]	DICOM Resolution [mm]
Study0	295 × 293	0.6
Study1	441 × 441	0.6
Study2	702 × 702	0.25
Study3	706 × 706	0.25
Study4	585 × 858	0.35
Study5	506 × 510	0.35

**Table 2:** Transformation parameters ranges which define the experiments.

Transformation ID	$t_{x}$ [px]	$t_{y}$ [px]	Angle [Deg]	Scale
T0	*rand*(−10:10)	*rand*(−10:10)	0	1
T1	0	0	*rand*(1:5)	1
T2	0	0	*rand*(30:45)	1
T3	*rand*(−10:10)	*rand*(−10:10)	*rand*(1:5)	1
T4	0	0	0	*rand*(1.1:1.5)
T5	0	0	0	*rand*(0.7:0.9)
T6	*rand*(−10:10)	*rand*(−10:10)	0	*rand*(1.1:1.5)
T7	*rand*(−10:10)	*rand*(−10:10)	0	*rand*(0.7:0.9)
T8	0	0	*rand*(1:5)	*rand*(1.1:1.5)
T9	0	0	*rand*(30:45)	*rand*(0.7:0.9)
T10	*rand*(−10:10)	*rand*(−10:10)	*rand*(1:5)	*rand*(1.1:1.5)
T11	*rand*(−10:10)	*rand*(−10:10)	*rand*(1:5)	*rand*(0.7:0.9)

## Data Availability

The data presented in this study are available on request from the corresponding author. The data are not publicly available due to privacy.
